# Circulating miRNAs associate with historical childhood asthma hospitalization in different serum vitamin D groups

**DOI:** 10.1186/s12931-024-02737-x

**Published:** 2024-03-08

**Authors:** Xiaoning Hong, Mingye Jiang, Alvin T. Kho, Anshul Tiwari, Haiyan Guo, Alberta L. Wang, Michael J. McGeachie, Scott T. Weiss, Kelan G. Tantisira, Jiang Li

**Affiliations:** 1 Clinical Big Data Research Center, The Seventh Affiliated Hospital of Sun Yat-Sen University, Shenzhen, Guangdong China; 2 Channing Division of Network Medicine, Brigham and Women’s Hospital, Harvard Medical School, Boston, MA USA; 3 Computational Health Informatics Program, Boston Children’s Hospital, Boston, MA USA; 4 Department of Respiratory and Critical Care Medicine, The Seventh Affiliated Hospital of Sun Yat-Sen University, Shenzhen, Guangdong China; 5 Shenzhen Key Laboratory for Systems Medicine in Inflammatory Disease, School of Medicine, Shenzhen Campus of Sun Yat-Sen University, Sun Yat-Sen University, Shenzhen, China; 6 Partners Personalized Medicine, Partners Healthcare, Boston, MA USA; 7 Department of Pediatrics, Division of Respiratory Medicine, University of California San Diego, La Jolla, CA USA; 8 Shenzhen Key Laboratory of Chinese Medicine Active Substance Screening and Translational Research, Shenzhen, Guangdong China

**Keywords:** miRNA, Circulating miRNA, Asthma, Childhood asthma, Vitamin D, Hospitalization, Exacerbation

## Abstract

**Background:**

Vitamin D may help to alleviate asthma exacerbation because of its anti-inflammation effect, but the evidence is inconsistent in childhood asthma. MiRNAs are important mediators in asthma pathogenesis and also excellent non-invasive biomarkers. We hypothesized that circulating miRNAs are associated with asthma exacerbation and modified by vitamin D levels.

**Methods:**

We sequenced baseline serum miRNAs from 461 participants in the Childhood Asthma Management Program (CAMP). Logistic regression was used to associate miRNA expression with asthma exacerbation through interaction analysis first and then stratified by vitamin D insufficient and sufficient groups. Microarray from lymphoblastoid B-cells (LCLs) treated by vitamin D or sham of 43 subjects in CAMP were used for validation in vitro. The function of miRNAs was associated with gene modules by weighted gene co-expression network analysis (WGCNA).

**Results:**

We identified eleven miRNAs associated with asthma exacerbation with vitamin D effect modification. Of which, five were significant in vitamin D insufficient group and nine were significant in vitamin D sufficient group. Six miRNAs, including hsa-miR-143-3p, hsa-miR-192-5p, hsa-miR-151a-5p, hsa-miR-24-3p, hsa-miR-22-3p and hsa-miR-451a were significantly associated with gene modules of immune-related functions, implying miRNAs may mediate vitamin D effect on asthma exacerbation through immune pathways. In addition, hsa-miR-143-3p and hsa-miR-451a are potential predictors of childhood asthma exacerbation at different vitamin D levels.

**Conclusions:**

miRNAs are potential mediators of asthma exacerbation and their effects are directly impacted by vitamin D levels.

**Supplementary Information:**

The online version contains supplementary material available at 10.1186/s12931-024-02737-x.

## Introduction

Asthma is the most common chronic disease in children characterized by airway inflammation in response to certain triggers, such as pollen, cold and other respiratory infections [1]. Asthma exacerbations are still the fourth leading cause of hospital stays among children aged 0–17 years with 100.7 stays per 100,000 in 2018 [2] and the direct costs of pediatric asthma were estimated at 6.31 billion in 2018 dollars [3].

Vitamin D is a fat-soluble secosteroid which can be synthesized by skin in the presence of ultraviolet B (UVB) radiation from sunlight or taken in from the supplementation [4]. Vitamin D is processed by the liver to form calcifediol (25(OH)D_3_) and then transported to kidney where 25(OH)D_3_ is converted to the active metabolite calcitriol (1,25(OH)_2_D_3_) [5]. Vitamin D is well-studied in regulating the level of calcium in the body and bone formation and also plays an important role in immune regulation and respiratory infections [6]. In our previous study, we associated 25-hydroxyvitamin D (25(OH)D) levels in serum at baseline with hospitalization or emergency department (ED) visits in the Childhood Asthma Management Program (CAMP). We confirmed a higher odds of hospitalization and ED visit for insufficient vitamin D status, but the mechanisms were still unclear [7].

MiRNAs are small non-coding RNAs with 18–25 nucleotides and act as post-transcriptional regulators in various biological processes and signaling pathways, such as cell apoptosis, immune and inflammation [8, 9]. MiRNAs also function in the asthma pathogenesis [10–12] and circulating miRNAs are good non-invasion biomarkers of asthma exacerbation, remission and ICS prognosis [13–16].

In this study, we investigated the association between miRNAs and asthma exacerbation as modified by serum vitamin D levels. We associated miRNA expression at baseline with historical asthma hospitalization in consideration of the interaction between miRNA and vitamin D first and then in vitamin D insufficient group and sufficient group separately. To investigate the potential pathways of the interaction effect that mediated by miRNAs, we performed microarray of lymphoblastoid B-cells treated by vitamin D or sham from 43 asthmatic subjects in CAMP to identified the differentially expressed genes (DEGs) and these DEGs were clustered into gene modules by WGCNA. These gene modules were enriched with functions by GO enrichment analysis and miRNAs with significant interaction effect were associated with these functional gene modules. We also demonstrate that these miRNAs can predict both historical and future hospitalizations at an excellent level.

## Methods

### Participant selection

We selected 461 subjects from the Childhood Asthma Management Program (CAMP) (Clinicaltrials.gov: NCT00000575 Date: October 27, 1999), which was a double-blind randomized controlled clinical trial [17, 18]. These participants provided serum samples at enrollment. The serum concentration of 25-hydroxyvitamin D3 was measured using a radioimmunoassay method in Dr. Bruce Hollis’ laboratory at the Medical University of South Carolina [19, 20] and then categorized into insufficient (≤ 30 ng/ml) and sufficient (> 30 ng/ml) [21]. The history of previous exacerbation was collected by questionnaires during the screening visits [18].

### Small RNA sequencing and profiling

We isolated total RNA of 461 subjects from serum samples using Qiagen miRNeasy Serum/Plasma extraction kit and QIAcube automation. All samples were quantified using the Nanodrop spectrophotometer prior to plating with the RNA concentration 30.97 ± 20.97 ng/μl (see Additional file 1: Fig. S1). We built the small RNA-Seq libraries by Norgen Biotek Small RNA Library Prep Kit and sequenced on the Illumina NextSeq 500 platform at 51 bp single end reads. The sequencing data was deposited in the Gene Expression Omnibus with the accession number GSE134897 [13]. COMPSRA was employed to evaluate the read quality and trim adapters [22]. Sequencing reads with quality score lower than 20 were removed. The qualified reads were aligned to human genome hg38 by STAR (v2.7.10b) [23] and miRNAs were annotated by COMPSRA on the basis of miRbase [24].

### Statistical analysis

Logistic regression was used to examine the association between miRNAs and the historical asthma hospitalization (Yes: Y = 1 and No: Y = 0). Age, sex, race and BMI were considered a priori as confounders in the multivariate logistic regression model. We investigated the interaction between miRNA and vitamin D groups and then examined the association in both the vitamin D insufficient group (Group = 1) and the sufficient group (Group = 0).

### Validation via functional module

We isolated lymphoblastoid B-cells (LCLs) from blood of 43 asthmatic subjects enrolled in the Childhood Asthma Management Program (CAMP) [25]. The LCLs were then cultured in RPMI 1640 medium supplemented with 5% fetal bovine serum, and 1× Penicillin/Streptomycin/l-Glutamine. Afterward, the cells were treated with a sham (culture media) and 1 μM of 1,25-OH vitamin D for 72 h. RNA was extracted from the cells using the RNA Miniprep column purification system provided by Stratagene (La Jolla, CA). Total RNA samples were profiled on the Illumina HT12 V4 microarray. Quantile normalization was used to normalize the gene expression. Differentially expressed genes (DEGs) were identified with P ≤ 0.05 [adjusted by Benjamini–Hochberg (BH) Procedure]. Weighted Gene Correlation Network Analysis (WGCNA) v1.72.1 was used to identify co-modulated gene modules and these modules were also associated with miRNAs [26]. GO enrichment analysis of the gene modules were conducted using R package “clusterProfiler” [27] and p values were adjusted by Benjamini–Hochberg (BH) Procedure.

### Validation via external dataset

We studied the dataset GSE106885 in Gene Expression Omnibus (GEO), which contains four asthma human bronchial epithelial cell samples and treated with calcitriol, poly I:C, both and sham (culture media) respectively [28]. The RNA-seq read files of samples treated with calcitriol and sham were downloaded from SRA (SRP124965) and taken as the external dataset. The raw reads were filtered by sequencing quality first than then aligned to human reference genome hg38 by STAR (v2.7.10b) [23]. The differentially expressed genes were calculated by edgeR (v3.40.0) [29] and p values were adjusted by Benjamini–Hochberg (BH) Procedure. The miRNA-target interactions comes from miRTarBase with strong evidence [30].

### Prediction

The random forest model was used to predict the historical asthma hospitalization and future asthma hospitalization during the first year CAMP clinical trial by baseline miRNA expression level. The R package “randomForest” was employed to conduct the prediction [31] and the area under receiver operating characteristic curve (AUROC) was used for evaluating model performance.

## Results

### Baseline characteristics

A total of 461 samples were investigated including 139 subjects in the vitamin D insufficient (30 ng/ml) group and 322 subjects in the vitamin D sufficient (> 30 ng/ml) group. The baseline characteristics are shown in Table 1. Subjects in the vitamin D insufficient group had older age (9.12 ± 2.16 vs. 8.74 ± 2.12 yr) and higher BMI (18.67 ± 3.69 vs. 17.82 ± 3.15 kg/m^2^). Race was significantly different between vitamin d insufficient and sufficient groups (P < 0.01). The sex and primary outcome of child ever in hospital for asthma was not significantly different between the vitamin D insufficient and sufficient groups.

**Table 1:** Childhood asthma management program (CAMP) subset study population characteristics

Characteristic	Vitamin D insufficient (n = 139)	Vitamin D sufficient (n = 322)	P value
Age, yr	9.12 ± 2.16	8.74 ± 2.12	0.08^*^
Sex			0.15^†^
Male	74 (53.2)	196 (60.9)	
Female	65 (46.8)	126 (39.1)	
Race			< 0.01^†^
White	84 (60.4)	278 (86.3)	
Black	49 (35.3)	30 (9.3)	
Hispanic	6 (4.3)	14 (4.3)	
BMI, kg/m^2^	18.67 ± 3.69	17.82 ± 3.15	0.02*
Household income			0.98^‡^
< $15,000	9 (6.5)	18 (5.6)	
$15,000-$29,000	21 (15.1)	50 (15.6)	
$30,000-$49,000	44 (31.7)	108 (33.8)	
> $50,000	61 (43.9)	134 (42.9)	
Decline response	4 (2.9)	9 (2.8)	
Don’t know	0 (0)	1 (0.3)	
Child ever in hospital for asthma			0.83^†^
Yes	46 (33.1)	103 (32.0)	
No	93 (66.9)	219 (68.0)	

Data presented as n (%) or mean ± SD

*BMI* Body Mass Index

*P value from Student’s t test

^†^P value from Chi-square test

^‡^P value from Fisher’s exact test

### Significant miRNAs in the vitamin D interactions analysis

We identified 11 miRNAs that were associated with historical hospitalization in the interaction analysis (nominal P ≤ 0.05, FDR ≤ 0.1) (see Table 2). The effect modification by vitamin D was observed in both positive and negative directions. Hsa-miR-24-3p had the strongest protective effect (OR = 0.31; P = 0.005) and hsa-miR-22-3p had the strongest risk effect (OR = 2.08; P = 0.02). Hsa-miR-146b-5p was the most significant miRNA (OR = 0.32; P = 0.001) and had a strong protective effect modification by vitamin D.

**Table 2:** Significant miRNAs in the serum vitamin D interactions analysis

miRNA	Univariate				Multivariate^a^			
	OR^b^	Lower CI	Upper CI	P Value^‡^	OR^b^	Lower CI	Upper CI	P value^‡^
hsa-miR-146b-5p	0.34	0.17	0.67	0.002	0.32	0.16	0.64	0.001
hsa-miR-192-5p	1.75	1.18	2.61	0.01	1.85	1.23	2.80	0.003
hsa-miR-24-3p	0.33	0.15	0.73	0.01	0.31	0.14	0.70	0.005
hsa-miR-451a	1.65	1.10	2.47	0.02	1.80	1.18	2.73	0.01
hsa-miR-143-3p	1.69	1.14	2.52	0.01	1.66	1.11	2.50	0.01
hsa-miR-151a-5p	0.51	0.31	0.85	0.01	0.53	0.32	0.90	0.02
hsa-miR-181a-5p	0.50	0.26	0.98	0.04	0.43	0.21	0.86	0.02
hsa-miR-22-3p	2.00	1.09	3.67	0.03	2.08	1.12	3.86	0.02
hsa-miR-423-3p	0.53	0.32	0.89	0.02	0.54	0.32	0.91	0.02
hsa-miR-16-5p	1.41	1.02	1.95	0.04	1.44	1.04	2.01	0.03
hsa-miR-146a-5p	0.54	0.29	1.00	0.05	0.51	0.27	0.96	0.04

^‡^P value of the interaction item miRNA*VD group

^a^Adjusted for age, sex, race and BMI

^b^OR of the interaction item miRNA*VD group

### Significant miRNAs in the stratified analysis

We examined the association between baseline miRNAs expression and dichotomized historical asthma hospitalization in a retrospective manner in both the vitamin D insufficient group and the sufficient group.

In the insufficient group, five miRNAs were nominally associated with risk of hospitalization in both univariate and multivariate analysis (nominal P ≤ 0.05, FDR ≤ 0.2) and the results were listed in Table 3. Three miRNAs (hsa-miR-24-3p, hsa-miR-423-3p and hsa-miR146b-5p) had the protective effect on asthma exacerbation, of which hsa-miR-24-3p had the smallest OR values (OR = 0.36; P = 0.005) in the multivariate models. Two miRNAs (hsa-miR-192-5p and hsa-miR-143-3p) had a higher risk effect on asthma exacerbation, of which hsa-miR-192-5p had the strongest OR values (OR = 1.52; P = 0.02) in the multivariate models. In addition, all the five miRNAs reported significant effect modification in the interaction analysis (see Table 2).

**Table 3:** Significant miRNAs in the serum vitamin D insufficient group

miRNA	Univariate				Multivariate^a^			
	OR	Lower CI	Upper CI	P Value	OR	Lower CI	Upper CI	P Value
hsa-miR-24-3p	0.38	0.19	0.75	0.01	0.36	0.18	0.73	0.005
hsa-miR-423-3p	0.59	0.39	0.88	0.01	0.59	0.39	0.89	0.01
hsa-miR-192-5p	1.44	1.07	2.07	0.03	1.52	1.08	2.15	0.02
hsa-miR-146b-5p	0.51	0.29	0.90	0.02	0.50	0.28	0.89	0.02
hsa-miR-143-3p	1.49	1.03	2.00	0.02	1.46	1.04	2.05	0.03

^a^Adjusted for age, sex, race and BMI

In the vitamin D sufficient group, nine miRNAs were associated with hospitalization in the univariate model and the association for each individual miRNA remains significant after adjusting for age, sex, race and BMI (nominal P ≤ 0.05, FDR ≤ 0.1) (see Table 4). Three miRNAs (hsa-miR-22-3p, hsa-miR-16-5p and hsa-miR-451a) were associated with a decreased risk of hospitalization, while six miRNAs (hsa-miR-125b-5p, hsa-miR-92b-3p, hsa-miR146b-5p, hsa-miR-423-5p, hsa-miR-125a-5p and hsa-miR-99b-5p) were associated with an increased risk of hospitalization. Hsa-miR-22-3p was associated with the lowest odds of hospitalization (OR = 0.62; P = 0.004) in the multivariate model and hsa-miR-99b-5p was associated with the highest odds of hospitalization (OR = 1.57; P = 0.05). Four miRNAs (hsa-miR-22-3p, hsa-miR-16-5p, hsa-miR-451a and hsa-miR-146b-5p) reported significant effect modification in the interaction analysis (see Table 2).

**Table 4:** Significant miRNAs in the serum vitamin D sufficient group

miRNA	Univariate				Multivariate^a^			
	OR	Lower CI	Upper CI	P value	OR	Lower CI	Upper CI	P value
hsa-miR-22-3p	0.60	0.44	0.84	0.003	0.62	0.44	0.86	0.004
hsa-miR-16-5p	0.78	0.64	0.94	0.01	0.78	0.64	0.95	0.01
hsa-miR-451a	0.77	0.60	0.97	0.03	0.75	0.59	0.96	0.02
hsa-miR-125b-5p	1.48	1.02	2.13	0.03	1.55	1.05	2.27	0.03
hsa-miR-92b-3p	1.28	1.02	1.61	0.03	1.30	1.03	1.64	0.03
hsa-miR-146b-5p	1.51	1.02	2.23	0.04	1.54	1.03	2.31	0.03
hsa-miR-423-5p	1.35	1.03	1.78	0.03	1.34	1.01	1.78	0.04
hsa-miR-125a-5p	1.50	1.04	2.16	0.04	1.47	1.01	2.14	0.04
hsa-miR-99b-5p	1.62	1.04	2.54	0.03	1.57	0.99	2.49	0.05

^a^Adjusted for age, sex, race and BMI

Hsa-miR-146b-5p was associated with hospitalization in both vitamin D insufficient (OR = 0.50; P = 0.02) and sufficient (OR = 1.54; P = 0.03) groups, as well as in the interaction analysis (OR = 0.32; P = 0.001), which implied potential function in asthma pathogenesis via vitamin D.

### Validate the function of miRNAs in the interaction analysis in vitro

**Fig. 1:**
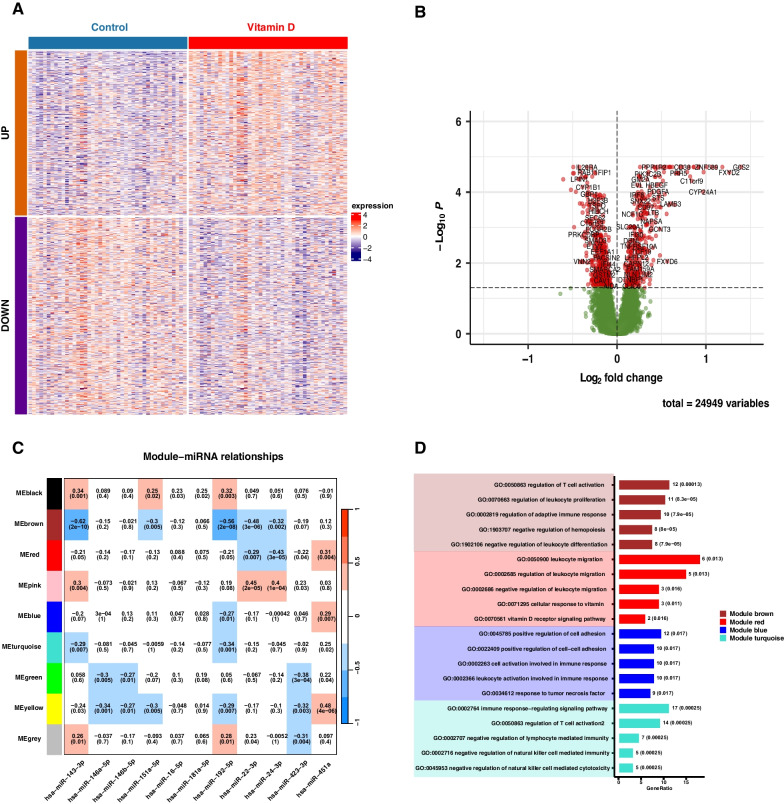
Identification of the differentially expressed genes (DEGs) between vitamin D treated LCLs and control in 43 CAMP participants. **A**, **B** Heatmap and Volcano plots show the DEGs. **C** Heatmap plot shows the relationships between the gene modules and 11 significant miRNAs in the interaction analysis in CAMP. **D** GO enrichment analysis of the turquoise, red, brown and blue gene modules (FDR < 0.05). *LCLs* lymphoblast-like B cells, *CAMP* Childhood Asthma Management Program

To validate whether the target genes of miRNAs that associated with hospitalization in the interaction analysis were functional at different vitamin D concentrations, we conducted microarray experiment from 43 participants in CAMP (see Additional file 1: Table S1). The lymphoblast-like B cells (LCLs) were isolated from blood and treated with 1,25-OH vitamin D or sham. We identified 811 genes that were differentially expressed between vitamin D treated LCLs and controls, of which 180 genes (22.19% of the differentially expressed genes) overlapped with the miRNA target genes (see Fig. 1A, B).

Based on the expression profiles of differentially expressed genes, we performed WGCNA analysis and identified 9 gene modules that were associated with at least one miRNA in the interaction analysis (see Fig. 1C). GO enrichment analysis was performed to annotate the function of these gene modules and we found that Module brown, Module red, Module blue and Module turquoise had significant biological processes (see Fig. 1D).

Module brown was enriched into leukocyte development related pathways (e.g. GO:1902106, negative regulation of leukocyte differentiation, FDR = 7.94 × 10^–5^) and negatively associated with five miRNAs, including hsa-miR-143-3p, hsa-miR-151a-5p, hsa-miR-192-5p, hsa-miR-22-3p and hsa-miR-24-3p. Of which, hsa-miR-143-3p (r = − 0.62; P = 2 × 10^–10^) had the largest absolute value of correlation coefficient. Module red was enriched into both vitamin D (e.g. GO: 0071295, cellular response to vitamin, FDR = 0.008) and leukocyte migration (e.g. GO:0050900, leukocyte migration, FDR = 0.009) pathways and associated with hsa-miR-22-3p, hsa-miR-24-3p and hsa-miR-451a. Of which, hsa-miR-24-3p (r = − 0.43; P = 3 × 10^–5^) had the strongest negative correlation. Module blue was enriched into both immune response (e.g. GO:0002366, leukocyte activation involved in immune response, FDR = 0.01) and cell adhesion (e.g. GO:0022409, positive regulation of cell–cell adhesion, FDR = 0.01) and associated with has-miR-192-5p and hsa-miR-451a. Module turquoise was also enriched into immune response (e.g. GO:0002764, immune response-regulating signaling pathway, FDR = 2.17 × 10^–4^) and associated with hsa-miR-143-3p and hsa-miR192-5p.

### Validate the function of miRNAs in the interaction analysis through external dataset

To gain a more comprehensive understanding of the effect modification between miRNAs and vitamin D, we download the dataset GSE106885 from Gene Expression Omnibus (GEO) which contains asthma human bronchial epithelial cell samples treated with calcitriol (bioactive vitamin D) and sham (culture media). We identified 1702 genes that were differentially expressed between the calcitriol treated and sham samples, of which 454 genes (26.7% of differentially expressed genes) were overlapped with the miRNA target genes (see Additional file 1: Figs. S2, S3). These overlapped genes were enriched into the signaling pathways about cell apoptosis, cell proliferation and immune function, which suggested that the effect modification of vitamin D on miRNAs may vary in different cell types (see Additional file 1: Fig. S4).

### Predict historical and future asthma hospitalization via baseline miRNAs expression

**Fig. 2:**
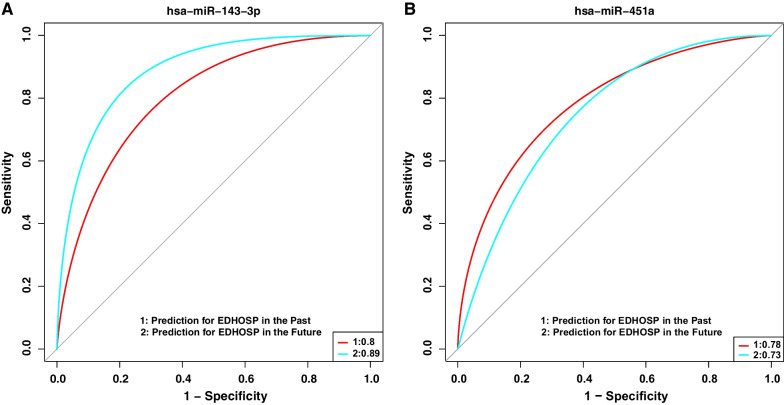
Prediction of historical and future asthma hospitalization via baseline miRNA expression in different vitamin D groups. The red curve denotes the prediction of historical asthma and the light blue curve denotes the prediction of asthma hospitalization in the first year of follow-up. **A** Prediction of miRNA hsa-miR-143-3p in vitamin D insufficient group (past: AUROC = 0.8; future: AUROC = 0.89). **B** Prediction of miRNA hsa-miR-451a in vitamin D sufficient group (past: AUROC = 0.78; future: AUROC = 0.73). *AUROC* area under receiver operating characteristic curve

We tried to predict the past asthma hospitalization via baseline miRNA expression in vitamin D insufficient and sufficient groups first and then examined that whether these miRNAs were able to predict the risk of future asthma hospitalization during the first year CAMP clinical trial. Among the five significant miRNAs in the vitamin D insufficient group (see Table 3), hsa-miR-143-3p performed the prediction best with AUROC 0.8 for the past asthma hospitalization and AUROC 0.89 for the future asthma hospitalization (see Fig. 2A). Among the nine significant miRNAs in the vitamin D sufficient group (see Table 4), hsa-miR-451a predicted best with AUROC 0.78 for the past asthma hospitalization and AUROC 0.73 for the future asthma hospitalization (see Fig. 2B). Both hsa-miR-143-3p and hsa-miR-451a had significant effect modification on vitamin D (see Table 2).

## Discussion

In our study, we identified eleven miRNAs in the interaction analysis, of which five miRNAs were associated with historical asthma hospitalization in the vitamin D insufficient group and nine miRNAs in the vitamin D sufficient group, suggesting miRNAs may be a pivotal vitamin D-related mediator of asthma severity in childhood asthma. These miRNAs were mainly associated with functional modules in immune system, such as leukocyte development, leukocyte migration, immune response, through microarray experiment in vitro. In addition, hsa-miR-143-3p and hsa-miR-451a could predict both historical and future hospitalization with good–excellent AUROC in vitamin D insufficient and sufficient groups.

Hsa-miR-146b-5p had a significant protective effect on childhood asthma in the vitamin D insufficient group and the significant risk effect in the vitamin D sufficient group. Bianchi and colleagues validated that hsa-miR-146b-5p was upregulated in T cells when treated with vitamin D [32]. Hsa-miR-146b-5p shared the same “seed” in sequence with hsa-miR-146a-5p which exhibited strong anti-inflammatory effect by down-regulating the expression of NF-κB [33, 34].

Hsa-miR-24-3p had the strongest protective effect in the vitamin D insufficient group and was associated with Module brown (leukocyte development) and Module red (leukocyte migration and vitamin D response) (see Fig. 1C, D). Lal et al. confirmed that miR-24-3p was consistently up-regulated in differentiated blood cells [35, 36]. Pua et al. reported that miR-24-3p could inhibit IL-4 production in T cells in vitro and alleviate allergic airway hypersensitivity inflammatory responses [37]. Xu et al. found that miR-24-3p could attenuate IL-1β induced chondrocyte injury associated with osteoarthritis [38]. MiR-24-3p also exerts anti-inflammatory function in LPS-stimulated macrophages by inhibiting pro-inflammatory cytokines [39].

Hsa-miR-22-3p had the strongest protective effect in the vitamin D sufficient group and was also associated with Module brown and Module red (see Fig. 1C, D). Lal et al. also reported that miR-22-3p was up-regulated when HL60 cells differentiating to monocytes using vitamin D3 [36]. Youn et al. confirmed the anti-inflammatory effect of miR-22-3p in macrophages [40]. Guo et al. validated that miR-22-3p was downregulated in the OVA-induced murine asthma model and LPS-induced bronchial epithelia cells, while overexpression of miR-22-3p ameliorated lung injury and inhibited epithelia cell injury [41]. Alvarez-Diaz and the group reported that miR-22-3p could be induced by 1,25(OH)_2_D_3_, which implied vitamin D had a strong effect on miRNA expression [42].

Hsa-miR-192-5p had the highest risk effect on asthma exacerbation in vitamin D insufficient group and was associated with Module brown, Module blue (immune response and cell adhesion) and Module turquoise (immune response) (see Fig. 1C, D). Zhang et al. showed miR-192-5p was associated with T follicular helper cell differentiation by regulating CXCR5 in childhood asthma [43] and Chu et al. found miR-192-5p could regulate the immune response by targeting Il-1RI [44].

The WGCNA reported that miRNAs interacting with vitamin D were associated with immune function in LCLs, while analysis of external dataset showed that these miRNAs were also associated with cell apoptosis and proliferation in bronchial epithelial cells. Both the validations suggest that vitamin D is a comprehensive regulator that may be involved in different functions in different cell types through interactions with miRNAs [45].

We attempted to use miRNAs to predict the past and future asthma hospitalization in both retrospective and prospective analysis through random forest method in different vitamin D groups. Hsa-miR-143-3p and hsa-miR-451a outperformed other miRNAs with the best AUROC, suggesting potential biomarkers of vitamin D related childhood asthma.

Our study had several limitations. First, we conducted our research in CAMP which is a clinical trial of childhood asthma, so the results should only be generalized to adult asthma with additional studies. Second, we lack a replication cohort for our results, but the validation for the WGCNA and enrichment analysis from microarray in vitro supports our main results.

## Conclusions

In summary, miRNAs are potential mediators and effect modifiers of asthma exacerbation as directly impacted by vitamin D levels.

### Supplementary Information

**Additional file 1: Table S1.** Demographic features of 43 subjects with asthma in LCLs microarray. **Figure S1.** RNA concentration of the subjects for sequencing in CAMP study. **Figure S2.** Volcano plots show the differentially expressed genes (DEGs) between the calcitriol treated and sham bronchial epithelial cell samples in GSE106885. **Figure S3.** Venn plot of target genes of 454 significant miRNAs in the interaction analysis and the differentially expressed genes in GSE106885. **Figure S4.** GO enrichment analysis of differentially expressed genes in dataset GSE106885.

## Data Availability

The datasets generated and/or analysed during the current study are available in the Gene Expression Omnibus (GSE134897) repository.

## Abbreviations

CAMP: Childhood Asthma Management Program
UVB: Ultraviolet B
ED: Emergency department
DEGs: Differentially expressed genes
WGCNA: Weighted Gene Correlation Network Analysis
LCLs: Lymphoblast-like B cells
BH: Benjamini–Hochberg
AUROC: Area under receiver operating characteristic curve
