# Supplementary material for: Circulating miRNAs associate with historical childhood asthma hospitalization in different serum vitamin D groups
Source: Respir Res. 2024 Mar 8;25:118. doi: 10.1186/s12931-024-02737-x (PMC10921757; doi:10.1186/s12931-024-02737-x)
Supplement: Supplementary file 1 — Additional file 1: Table S1. Demographic features of 43 subjects with asthma in LCLs microarray. Figure S1. RNA concentration of the subjects for sequencing in CAMP study. Figure S2. Volcano plots show the differentially expressed genes (DEGs) between the calcitriol treated and sham bronchial epithelial cell samples in GSE106885. Figure S3. Venn plot of target genes of 454 significant miRNAs in the interaction analysis and the differentially expressed genes in GSE106885. Figure S4. GO enrichment analysis of differentially expressed genes in dataset GSE106885. [file 12931_2024_2737_MOESM1_ESM.docx]

**Circulating MiRNAs Associate with Historical Childhood Asthma Hospitalization in Different Serum Vitamin D Groups**

Xiaoning Hong ^1^, Mingye Jiang ^1^, Alvin T. Kho ^2,3^, Anshul Tiwari^2^, Haiyan Guo^4,5^, Alberta L. Wang^2^, Michael J. McGeachie^2^, Scott T. Weiss ^2,6^, Kelan G. Tantisira ^2,7,^* and Jiang Li ^1,2,8,^*

^1^Clinical Big Data Research Center, The Seventh Affiliated Hospital of Sun Yat-Sen University, Shenzhen, Guangdong, China;

^2^Channing Division of Network Medicine, Brigham and Women’s Hospital, Harvard Medical School, Boston, MA, USA;

^3^Computational Health Informatics Program, Boston Children’s Hospital, Boston, MA, USA;

^4^Department of Respiratory and Critical Care Medicine, The Seventh Affiliated Hospital of Sun Yat-Sen University, Shenzhen, Guangdong, China;

^5^Shenzhen Key Laboratory for Systems Medicine in Inflammatory Disease, School of Medicine, Shenzhen Campus of Sun Yat-Sen University, Sun Yat-Sen University, Shenzhen, China.

^6^Partners Personalized Medicine, Partners Healthcare, Boston, MA, USA;

^7^Department of Pediatrics, Division of Respiratory Medicine, University of California San Diego, La Jolla, CA, USA;

^8^Shenzhen Key Laboratory of Chinese Medicine Active Substance Screening and Translational Research, Guangdong, Shenzhen, China;

*Correspondence: [ktantisira@health.ucsd.edu](mailto:ktantisira@health.ucsd.edu) (K.G.T.); Tel.: +1-(858)-966-5846; [lijiang29@mail.sysu.edu.cn](mailto:lijiang29@mail.sysu.edu.cn) (J.L.); Tel.: +86-(0755)-81206999

**Table S1.** Demographic features of 43 subjects with asthma in LCLs microarray.

|  | **Vitamin D Sufficient**  **(N=26)** | **Vitamin D Insufficient**  **(N=17)** | **P** |
| --- | --- | --- | --- |
| **Age, yr.** | 8.42 (1.94) | 8.42 (2.09) | 0.999^*^ |
| **Sex** |  |  | 0.256^#^ |
| **Male** | 18 (69.2) | 8 (47.1) |  |
| **Female** | 8 (30.8) | 9 (52.9) |  |
| **Race** |  |  |  |
| **White** | 26 (100.0) | 17 (100.0) | - |
| **Vitamin D, ng/ml** | 53.90 (16.02) | 25.52 (3.14) | <0.001^*^ |

^*^P value comes from Student’s t test.

^#^P value comes from Chi-square test.

**Figure S1.** RNA concentration of the subjects for sequencing in CAMP study.


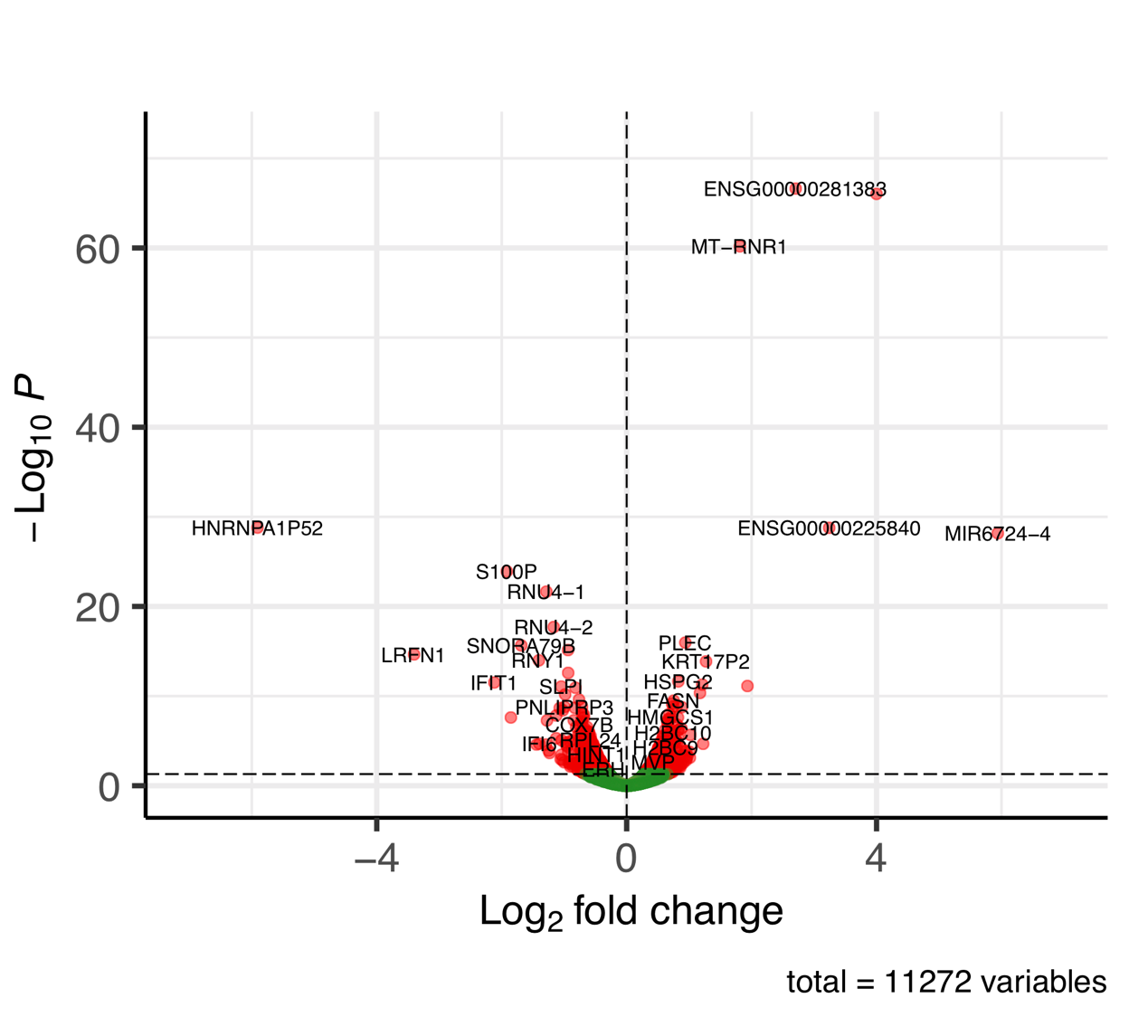


**Figure S2.** Volcano plots show the differentially expressed genes (DEGs) between the calcitriol treated and sham bronchial epithelial cell samples in GSE106885.

**Figure S3.** Venn plot of target genes of 454 significant miRNAs in the interaction analysis and the differentially expressed genes in GSE106885.

**Figure S4.** GO enrichment analysis of differentially expressed genes in dataset GSE106885.
